# Formulation of Indomethacin Colon Targeted Delivery Systems Using Polysaccharides as Carriers by Applying Liquisolid Technique

**DOI:** 10.1155/2014/704362

**Published:** 2014-05-26

**Authors:** Kadria A. Elkhodairy, Hanna A. Elsaghir, Amal M. Al-Subayiel

**Affiliations:** ^1^Department of Pharmaceutics, College of Pharmacy, King Saud University, P.O. Box 22452, Riyadh 11495, Saudi Arabia; ^2^Department of Industrial Pharmacy, Faculty of Pharmacy, Alexandria University, Alexandria, Egypt; ^3^Department of Pharmaceutics, Faculty of Pharmacy, Cairo University, Cairo, Egypt

## Abstract

The present study aimed at the formulation of matrix tablets for colon-specific drug delivery (CSDD) system of indomethacin (IDM) by applying liquisolid (LS) technique. A CSDD system based on time-dependent polymethacrylates and enzyme degradable polysaccharides was established. Eudragit RL 100 (E-RL 100) was employed as time-dependent polymer, whereas bacterial degradable polysaccharides were presented as LS systems loaded with the drug. Indomethacin-loaded LS systems were prepared using different polysaccharides, namely, guar gum (GG), pectin (PEC), and chitosan (CH), as carriers separately or in mixtures of different ratios of 1 : 3, 1 : 1, and 3 : 1. Liquisolid systems that displayed promising results concerning drug release rate in both pH 1.2 and pH 6.8 were compressed into tablets after the addition of the calculated amount of E-RL 100 and lubrication with magnesium stearate and talc in the ratio of 1 : 9. It was found that E-RL 100 improved the flowability and compressibility of all LS formulations. The release data revealed that all formulations succeeded to sustain drug release over a period of 24 hours. Stability study indicated that PEC-based LS system as well as its matrix tablets was stable over the period of storage (one year) and could provide a minimum shelf life of two years.

## 1. Introduction

Colonic drug delivery has gained increased importance not only for the delivery of the drugs for the treatment of local diseases associated with the colon, such as Chron's diseases, ulcerative colitis, colorectal cancer, and amebiasis, but also for its potential for the delivery of proteins and therapeutic peptides [1].

Natural polysaccharides are now extensively used for the development of solid dosage forms for delivery of drug to the colon. The rationale for the development of a polysaccharide based delivery system for colon is the presence of large amounts of polysaccharides in the human colon as the colon is inhabited by a large number and variety of bacteria which secrete many enzymes, for example, *β*-d-glucosidase, *β*-d-galactosidase, amylase, pectinase, xylanase, *β*-d-xylosidase, and dextranase. A large number of polysaccharides have already been studied for their potential as colon-specific drug carrier systems, such as chitosan, pectin, chondroitin sulphate, cyclodextrin, dextrans, guar gum, inulin, amylose, and locust bean gum [2].

Successful delivery to the colon requires the drug to be in solution form before it arrives at the colon or, alternatively, it should dissolve in the luminal fluids of the colon, but this can be a limiting factor for poorly soluble drugs as the fluid content in the colon is much lower than in the upper part of the gastrointestinal (GI) tract. In addition, because of the high water absorption capacity of the colon, the colonic contents are considerably viscous and their mixing is not efficient; thus, availability of most drugs to the absorptive membrane is low. Furthermore, the stability of the drug is also a concern and must be taken into consideration while designing the delivery system. The drug could potentially bind in a nonspecific manner to dietary residues, intestinal secretions, mucus, or fecal matter [3, 4].

An important factor that must be considered in designing and formulation of therapeutic dosage form is the aqueous solubility of a drug substance. A drug must possess some aqueous solubility for therapeutic efficacy. For a drug to enter the systemic circulation and exert a therapeutic effect, it must first be in solution. Relatively insoluble compounds often exhibit incomplete or erratic absorption. If the solubility of the drug substance is less than desirable, consideration must be given to improve its solubility and dissolution rate [5]. The methods to accomplish this depend on the chemical nature of the drug and the type of drug product under consideration. Various techniques are reported to improve the dissolution of poorly soluble drugs, including solid dispersions [6], crystal engineering [7], ball milling [8], complexation [9], self-emulsifying drug delivery systems [10], and the use of mesoporous silica carriers [11].

The liquisolid technique [12] has shown promise for improved dissolution rate of poorly water-soluble drugs. The liquisolid technique as described by Spireas [13] is a novel concept, where a liquid may be transformed into a free flowing, readily compressible, and apparently dry powder by simple physical blending with selected carrier and coating material [13, 14]. The liquid portion, which can be a liquid drug, a drug suspension, or a drug solution in suitable nonvolatile liquid vehicles (liquid medication), is included into the porous carrier material. As the carrier is saturated with liquid, a liquid layer is formed on the particle surface which is instantly adsorbed by the fine coating particles [15, 16]. The liquisolid compacts are acceptably flowing and compressible powdered forms of liquid medications.

In the liquisolid systems (LSS), even though the drug might be in a solid dosage form, it is held within the powder substrate in solution or in a solubilized, almost molecularly dispersed state [17, 18]. Therefore, due to their significantly increased wetting properties and surface area of drug available for dissolution, liquisolid compacts of water-insoluble substances may be expected to display enhanced drug release characteristics and consequently improved oral bioavailability [18–20].

The formulation of poorly water-soluble drugs into different drug delivery systems is usually performed in two steps. The first step is the preformulation study to increase its solubility using an appropriate solubilization technique and hence its oral bioavailability. The second step is the formulation of the required drug delivery system. Thus, the preparation of colon targeted drug delivery system of poorly soluble drug using liquisolid technique could exclude the step of preformulation study and the colon drug delivery system could be prepared directly in one step. The application of liquisolid technique to prepare colon targeted drug delivery system could be considered effective in presenting the drug in a soluble form at the site of absorption, thus overcoming the problem of the low fluid and high viscosity of the colon content.

Indomethacin is a nonsteroidal anti-inflammatory drug (NSAID) used most commonly for the treatment of inflammation and pain resulting from rheumatic arthritis. Indomethacin has anticolorectal cancer activity through cyclooxygenase- (COX-) independent mechanisms [21–23]. In spite of its benefits, indomethacin appears to have a high prevalence of gastric side effects [24]. Therefore, indomethacin is considered as a good candidate for colon delivery.

This study aimed at designing and formulation of matrix tablets for colon-specific drug delivery (CSDD) system of IDM by applying liquisolid technique. Liquisolid systems of IDM were prepared using different polysaccharides, namely, GG, PEC, and CH, as carriers separately or in mixtures of different ratios of 1 : 3, 1 : 1, and 3 : 1. Then a CSDD system based on time-dependent polymethacrylates and enzyme degradable polysaccharides was established. Eudragit RL 100 was employed as time-dependent polymer whereas liquisolid systems loaded with IDM were used as they presented the bacterial degradable polysaccharides. Liquisolid systems that showed promising results concerning drug release rate were compressed into tablets after the addition of the calculated amount of E-RL100 to give IDM matrix tablets for colon delivery.

## 2. Experimental

### 2.1. Materials

Indomethacin (*γ*-polymorphic form) (IDM) was kindly supplied by Pharco Pharmaceuticals, Alexandria, Egypt, guar gum (GG) by Premcem Gums Ltd., India, pectin (PEC) by BDH Co., England, chitosan (CH) by Sigma-Aldrich, Germany, microcrystallinecellulose (Avicel pH 102) by FMC Co., USA, colloidal silicone dioxide (Aerosil 200) by FMC Co., Philadelphia, PA, USA, Eudragit RL 100 (E-RL100) by Rhone-Pharma, Germany, polyethylene glycol 400 (PEG 400) by European Co. For Pharmaceutical Industries, Egypt, propylene glycol (PG) and polysorbate 80 (Tween 80) by Fisher, United Kingdom, and glycerin by Parchem Fine & Specialty Chemicals, New York, USA. All other chemicals and buffers were of analytical reagent grades.

### 2.2. Solubility Studies

To select the best nonvolatile solvent for dissolving or suspending IDM in liquid medication, solubility studies were performed according to the method of Higuchi and Connors [25]. The solubility of IDM was determined in different four nonvolatile solvents, namely, PEG 400, Tween 80, PG, and glycerin. Saturated solutions were prepared by adding excess drug to the vehicles in screw-capped vials. The suspensions were shaken in a thermostatically controlled water bath at 37 ± 0.5°C for 24 hours under constant vibration. The solutions were filtered through a 0.45 *μ*m Millipore filter, diluted with phosphate buffer (PB) pH 6.8, and analyzed spectrophotometrically at a wavelength of 318 nm against blank sample (blank sample contained the same concentration of specific solvent used without drug).

### 2.3. Calculation of the Loading Factors

In this study, PEG 400 was used as the liquid vehicle, Avicel PH 102 and different polysaccharides, namely, GG, PEC, and CH, as the carriers, and Aerosil 200 as the coating material, respectively. The carrier materials were used separately or in mixtures of different ratios (3 : 1, 1 : 1, and 1 : 3). In order to address the flowability and compressibility of LS systems, simultaneously, the “new formulation mathematical model of liquisolid systems” was employed [12, 26].

To calculate the loading factor, the liquid medication without drug which is the nonvolatile solvent PEG 400 was added to 20 gram of Avicel PH 102 and blended for 10 min (reference formulae). The same procedure was followed to calculate the loading factors for different LSS using the different polysaccharides as carriers and mixtures of different carriers in appropriate ratios. The loading factors for each formulation in the appropriate ratios were obtained by using Lf = *W*/*Q* formula, where *W* is the weight of liquid medication and *Q* is the amount of the carrier [12, 26]. The loading factors of eighteen LSS of different composition were determined.

### 2.4. Preparation of the Liquisolid Systems

The calculated amount of IDM was accurately weighed and dissolved in the determined volume of PEG 400. The resultant liquid medication was added to 20 gm of the carrier in a porcelain dish and blended for 10 min. The appropriate amount of the coating material was then added. Mixing process was carried out in three steps as described by Spireas and Bolton [26]. During the first stage, the system was blended at an appropriate mixing rate of one rotation per second for approximately one minute in order to evenly distribute liquid medication in the powder. In the second stage, the liquid/powder admixture was evenly spread as a uniform layer on the surfaces of mortar and left standing for approximately 5 min to allow drug solution to be absorbed in the interior of powder particles. In the third stage, powder was spread off the mortar surfaces by means of aluminum spatula. Eighteen LSS of different composition were prepared and evaluated. Only six systems were selected for further studies (Table 1).

### 2.5. Evaluation of the Prepared Liquisolid Systems

#### 2.5.1. Fourier Transform-Infrared (FT-IR) Study

FT-IR spectra of physical mixtures (1 : 1) of IDM and various excipients, as well as the different LSS, were performed to find out any possible drug-excipients interaction by KBr disc method using Perkin-Elmer FT-IR series spectrophotometer at a pressure of 90 psi for 5 min over the range 4000–450 cm^−1^.

#### 2.5.2. Drug Content

An amount of the prepared LSS (250 mg) was weighed accurately and triturated with 5 mL of ethanol, and finally the volume was made up to 100 mL with the PB pH 6.8. The solution was filtered through a Millipore membrane filter (0.45 *μ*m). The filtrate was used to determine the drug content using UV spectrophotometer at 318 nm. Each sample was analyzed in triplicate. Theoretically, each 250 mg of LSS should contain 25 mg of IDM (drug content should be 10 w/w).

#### 2.5.3. Flowability Studies

In order to ensure the flow properties of the LSS that will be selected to be compressed into tablets, angle of repose measurements (fixed height cone method), Carr's index, and Hausner ratio were adopted [27]. In the bulk density measurement, fixed weight of each of the prepared liquisolid powder formulae was placed in graduated cylinder and the volume (V0) occupied was measured and the initial bulk density (D0) was calculated. The graduated cylinder was then tapped at a constant velocity till a constant volume is obtained when the powder is considered to reach the most stable arrangement; the volume of the powder was then recorded as the final bulk volume (Vf), and then the final bulk density (Df) was calculated.

Carr's compressibility index was calculated according to the following equation:

(1) Carr's  index%=Df−D0Df×100.  

In addition, Hausner ratio was calculated from the following equation:

(2) Hausner's  ratio=DfD0.

The procedure was done in triplicate. Angle of repose, Carr's compressibility index, and Hausner ratio with the corresponding standard deviations for each of the prepared formulae were calculated (Table 2).

#### 2.5.4. Particle Size Analysis

Particle size of the different LS formulae was determined using the sieving method. A set of sieves (710–250 *μ*m) was used. The prepared LSS was placed on the top sieve (710 *μ*m) and placed into the sieve shaker and shaking was performed for 10 min. The amount retained on each sieve was weighed. Then the percentage of weight fraction retained on each sieve was calculated. The results were plotted as histograms to show the particle size distribution of each formula under investigation.

#### 2.5.5. In Vitro Dissolution Studies

The dissolution rate of IDM from LS systems was determined using USP XXIV dissolution rate apparatus II at a stirring rate of 100 ± 2 and containing 750 mL of 0.1 N HCl (pH 1.2) at 37 ± 0.5°C. An amount of each LS formula containing an equivalent of 25 mg IDM filled in a gelatin capsule (size 1) was placed in the dissolution medium. At predetermined time intervals, 5 mL aliquot was withdrawn and immediately was replaced with an equal volume of dissolution medium at the same temperature. The aliquot withdrawn was filtered through 0.45 *μ*m Millipore membrane filter, diluted adequately, and analyzed spectrophotometrically for their IDM content at *λ*_max⁡_ 318 nm against a blank. The same procedure was repeated for each formula using 750 mL of PB pH 6.8 for further 4 hours. The experiments were done in triplicate for each of the selected LSS and for control capsule containing 25 mg of IDM.The calculation of dissolution efficiency (DE) has been applied for the evaluation of pharmaceutical equivalence between LSS [28].

### 2.6. Preparation of Compressed Tablets of Liquisolid Systems

The selected LSS were used for the preparation of controlled release colon targeted tablets using E-RL 100.

#### 2.6.1. Fourier Transform-Infrared Spectroscopy (FT-IR) Study

FT-IR spectra of physical mixtures (1 : 1) of IDM and various excipients including E-RL 100, as well as the different LS systems and excipients used in preparation of matrix tablet, were performed to find out any possible drug-excipients interaction by KBr disc method using Perkin-Elmer FT-IR series spectrophotometer.

#### 2.6.2. Flow Characteristics of Tablet Formulations

The flow properties of mixtures of tablet components were studied. Angle of repose, Carr's index, and Hausner ratio were determined as mentioned before. The same procedure was repeated after the addition of the appropriate amount of (Mg.st) : (talc) mixture in the ratio of 1 : 9.

#### 2.6.3. Preparation of IDM Tablets

Mixtures of the prepared LSS and E-RL 100 in the ratio of 2 : 1 were prepared by adding the calculated amount of E-RL 100 geometrically to each of the selected LS systems, namely, F2, F3, F4, F5, and F6, and then mixed thoroughly for 10 minutes. A weighed amount of the lubricant (3% w/w of the LSS-E-RL mixture) was then added and mixed thoroughly for another 10 minutes. Then the powder mixture was compressed into tablet using a single punch tablet press with 12 mm punch and die at a pressure of 10–12 Kg/cm^2^. The tablet weight was varied from 435 to 450 mg depending on the drug content of each system. Each tablet was prepared to contain an amount of LS system equivalent to 25 mg of IDM. Therefore, two tablets of each formulation were used in the dissolution study (Table 4). Indomethacin conventional compressed tablets were prepared by direct compression of the physical mixtures of the drug, carrier, coat, E-RL 100, and lubricant. The conventional tablets were prepared as reference formulae for comparison (Table 5).

### 2.7. Evaluation of the Prepared Tablets

#### 2.7.1. Drug Content

IDM content in different LS tablet formulations was determined by accurately weighing 10 tablets of each formula individually. Each tablet was then crushed and dissolved in 100 mL PB pH 6.8, and then the solution was filtered, properly diluted, and then measured spectrophotometrically at *λ*_max⁡_ of 318 nm; thereafter, IDM content of each tablet was determined.

#### 2.7.2. Morphological Examination of Tablet

A tablet was placed in a Petri dish and immersed in 75 mL 0.1 N HCl solution (pH 1.2). The liquid was removed carefully after 2 hours with a syringe and a photograph of the tablet was taken with a digital camera fitted with zoom lens (NIKON COOLPIX L18, Japan). The same tablet was again immersed in 75 mL of PB pH 6.8. Liquid was then removed after 2 hours and a photograph of the tablet was taken. Tablet diameter was measured and the average of three measurements was considered. Further study was conducted using F2 matrix tablet to emphasize the swelling of the matrix along the vertical axis. The matrix tablet was placed in a beaker containing 35 mL of dissolution medium. The test was carried out in pH 1.2 for 2 hours then the matrix was transferred to pH 6.8 and the swelling behavior was recorded for 24 hours.

#### 2.7.3. In Vitro Dissolution Rate

Dissolution rate of IDM from LS matrix tablets was determined using USP XXIV dissolution rate apparatus I (Pharma Test, Germany) at a stirring rate of 100 ± 2 and containing 750 mL of 0.1 N HCl (pH 1.2) at 37 ± 0.5°C for 2 hours then the medium was rendered alkaline to pH 6.8 by the addition of the calculated amount of dihydrogen sodium phosphate. The dissolution studies at pH 6.8 were conducted for further 24 hours. Two tablets of each formula, containing an equivalent of 50 mg IDM, were placed in the dissolution medium. At predetermined time intervals, aliquot of 5 mL from the dissolution medium was withdrawn and immediately replaced with an equal volume of preheated fresh dissolution medium at 37 ± 0.5°C. The aliquots withdrawn were filtered through 0.45 *μ*m Millipore membrane filter, adequately diluted, and analyzed spectrophotometrically for their IDM content at *λ*_max⁡_ 318 nm using UV-Spectrophotometer (Genesys TM 5, Thermospectronic, USA). The experiments were done in triplicate for each of the selected LSS. The mean percentage cumulative amount of the drug dissolved was plotted against time.

#### 2.7.4. Preparation of Mimic Enzymatic Media of the Colon (Rat Cecal Matter (RCM))

A group of 5 male Albino Wister rats each weighing 150–200 g and maintained on normal diet (soaked grain of polysaccharide) was used to induce enzymes specifically acting on each polysaccharide used. The rats were then killed using CO_2_ asphyxiation, 45 minutes before the study. The abdomen was opened and the cecal was traced, legated at both ends, dissected, and immediately transferred into PB pH 6.8, previously bubbled with CO_2_. The cecal bags were opened; their contents individually were weighed, pooled, and then suspended in pH 6.8 to give 2% w/v dilution. As the cecum is naturally anaerobic, all operations were carried out under CO_2_ [29]. The procedure was performed in accordance with the guidelines of the local institutional animal ethics committee.

#### 2.7.5. In Vitro Drug Release in Rat Cecal Matter

Drug release studies in the presence of rat cecal content were also carried out using USP dissolution test apparatus I but with slight modification. After completing the test in pH 1.2 for 2 hours and pH 7.4 for 3 hours, basket containing tablets was immersed in 250 mL beaker containing PB solution (pH 6.8) and rat cecal content was maintained in the jars of the dissolution apparatus for up to 24 hours. Samples of 5 mL each were withdrawn at different time intervals (6, 7, 8, 9, and 24 hours), filtered using filter paper, and assayed spectrophotometrically for IDM at 318 nm. The same volume of fresh medium bubbled with CO_2_ was added after each withdrawn sample [30].

### 2.8. Drug Release Kinetics

In order to investigate the mechanism of drug release from compressed tablets, the release data were fitted into first-order, zero-order, Higuchi models, and the Korsmeyer model [31].

### 2.9. Stability on Storage

Storage test was performed on LSS selected for preparation of compressed tablets. Liquisolid systems and their corresponding matrix tablet formulations were kept on shelf for 9–12 months (to study the effect of fluctuation in temperature and humidity on the proposed formulations). The different temperatures and relative humidity (RH) under which this study was carried out were in the range of 25–45°C and 10–16%. These conditions are those of area where the formulations were prepared and would be stored and sold. All formulations were tested for any changes in physical appearance, drug content, in vitro release profiles, and any chemical interactions by FT-IR analysis.

### 2.10. Statistical Analysis

All the results were expressed as mean values ± standard deviation (SD). One way analysis of variance (ANOVA) with Tukey's multiple comparisons post hoc (Graph-Pad prism 6 program) was used to test for significance, at a 5% significance level. Statistical difference dealing (*P* < 0.05) was considered significant.

## 3. Results and Discussion

### 3.1. Solubility Studies

Solubility studies revealed that the solubility of IDM can be improved in presence of PEG 400. It was found that solubility of IDM increased to 95.53 mg/mL in PEG 400. A higher fraction of the drug in PEG 400 is in the molecular state and this would help to increase dissolution rate of the drug because the drug is already dissolved [32].

### 3.2. Determination of the Loading Factor

Composition of different LSS and their loading factors are summarized in Table 1. The loading factors varied from 0.840 (F1) to 0.887 (F3), respectively, in absence of the drug. However, upon the addition of IDM, the loading factors increased because the weight of liquid medication increased. It has been reported that, in conventional LS formulation, it is difficult to prepare formulation with good flowability and compatibility when loading factor is above 0.25 [12]. In this study, as the prepared LSS showed loading factors above 0.25, they were considered unacceptable formulations with respect to flowability and compatibility.

### 3.3. Evaluation of the Prepared Liquisolid Systems

#### 3.3.1. Fourier Transform-Infrared Spectroscopy (FT-IR) Study

**Figure 1:**
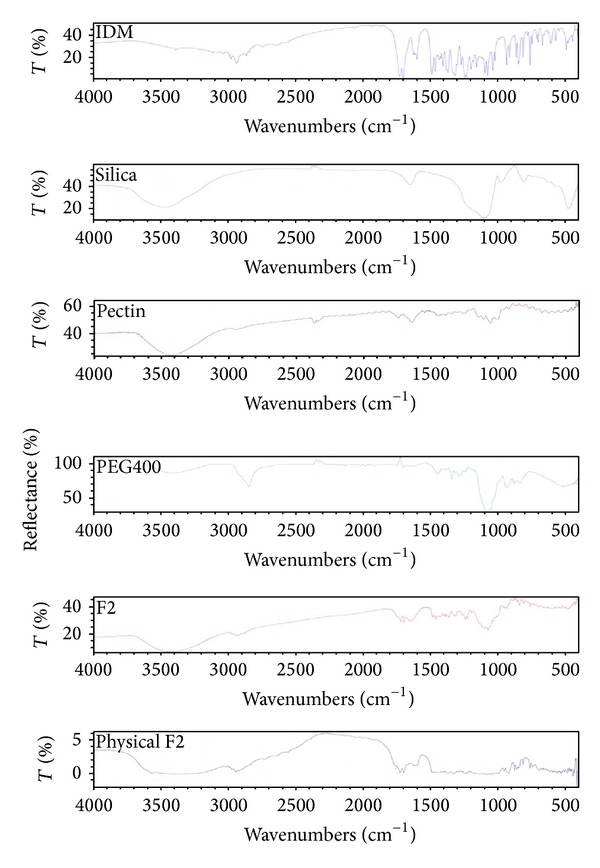
FTIR spectra of IDM, PEC, silica, PEG 400, liquisolid system, and physical mixture of formulation F2.

The FT-IR spectra of selected formulations were determined and only one spectrum of a representative formulation is included. FT-IR spectrum of PEC-based LS system as well as PEC-based physical mixture is presented in Figure 1. The characteristic peaks of the pure drug were compared with peaks obtained from the respective LSS (F2). It was observed that characteristic peaks of IDM appear with identical or with minor differences, at frequencies of 3370.33 and 1717.14 cm^−1^ corresponding to carboxylic O−H and C=O stretch, respectively. The spectrum shows also characteristic peaks at 2961.65 cm^−1^ (C–H stretching vibrations), 1691.83 cm^−1^ (C=O stretching vibrations), 1234.15 cm^−1^ (asymmetric aromatic O–C stretching), 1086.55 cm^−1^ (symmetric aromatic O–H stretching), and 1479.50 cm^−1^ (C–C stretching) [33].

FT-IR spectrum of PEC shows characteristic peaks at 3421.68 cm^−1^ and 2932 cm^−1^, which is due to the presence of –OH and –CH stretching vibration peaks, respectively. The peaks observed at 1735.19 cm^−1^ can be assigned to carboxymethyl (−COOCH3) peaks of PEC. The peaks at 1437.66 and 1357 cm^−1^ could be assigned to CH_2_ and –OH bending vibration peaks, respectively. The spectrum shows O–H bending at 1052.59 cm^−1^, C=O stretching at 1637.03 cm^−1^, and C–H stretching at 2361.07 cm^−1^ [34]. It can be seen that the peaks of the LSS F2 as well as F2 physical mixture are the sum of the characteristic peaks of the drug and the corresponding excipients used. The spectra of Figure 1 indicated the compatibility between the drug and each excipient used for preparation of LSS F2. The same results were obtained with the other prepared LSS (F1, F3, F4, F5, and F6) indicating no drug excipient interactions.

#### 3.3.2. Drug Content

The results of the drug content studies revealed that the amount of the drug present in 250 mg LSS varied from 18.54 ± 0.028 mg (7.42%) for F1 to 19.67 ± 0.037 mg (7.87%) for F3.

#### 3.3.3. Flow Properties

Liquisolid systems F1, F2, F4, F5, and F6 showed range of angle of repose; the lowest value was 34.00 (F1) and the highest value was 40.91 (F3). These values indicated that these formulations were LSS with good to fair flowability according to the USP [35]. Liquisolid systems F3 and F6 showed angles of repose of 40.91° and 40.70°, thus, exhibiting passable flow characteristics [35]. In addition, Hausner ratios and Carr's index of LS systems F2–F6 were higher than 1.250 and 21.00%, respectively, indicating fair flow characteristics [27]. Formulation F1 (GG-based LS) had Hausner ratio and Carr's index of 19.70% and 1.29, respectively, indicating good flow properties (Table 2).

#### 3.3.4. Particle Size Analysis

In general, the highest percentage of the weight fraction of the particles was found in the particle size range of 250–500 *μ*m. The lowest percentage of weight fraction of the coarser particles was found in the particle size range of 500–600 *μ*m except for F3 that showed high% of weight fraction in this range. Formulation F4 showed nearly equal percentage of weight fraction of the particles in the ranges of 250–500 and 600–710 *μ*m. Depending on the results of particle size analysis, average particle size of 375 *μ*m was used for further evaluation.

#### 3.3.5. In Vitro Dissolution Studies

**Figure 2:**
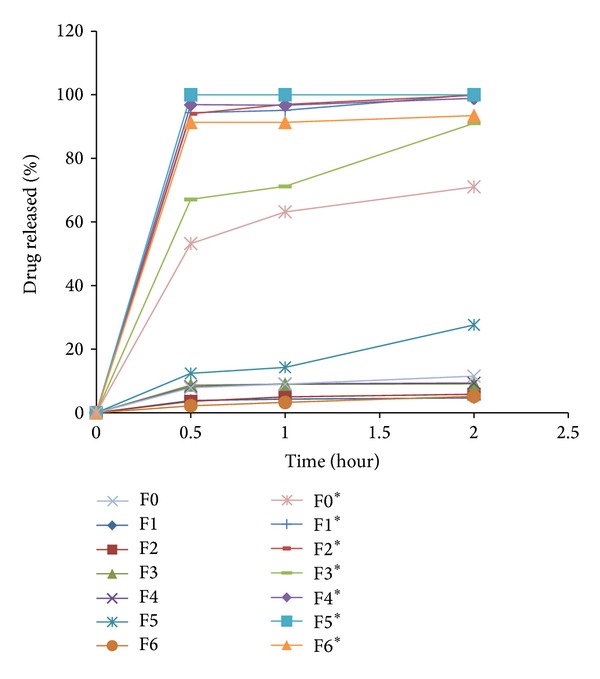
Release profiles of IDM from different liquisolid formulations (F1–F6) and pure drug (F0) in 0.1 N HCl (F) and in PB pH 6.8 (F*).

**Figure 3:**
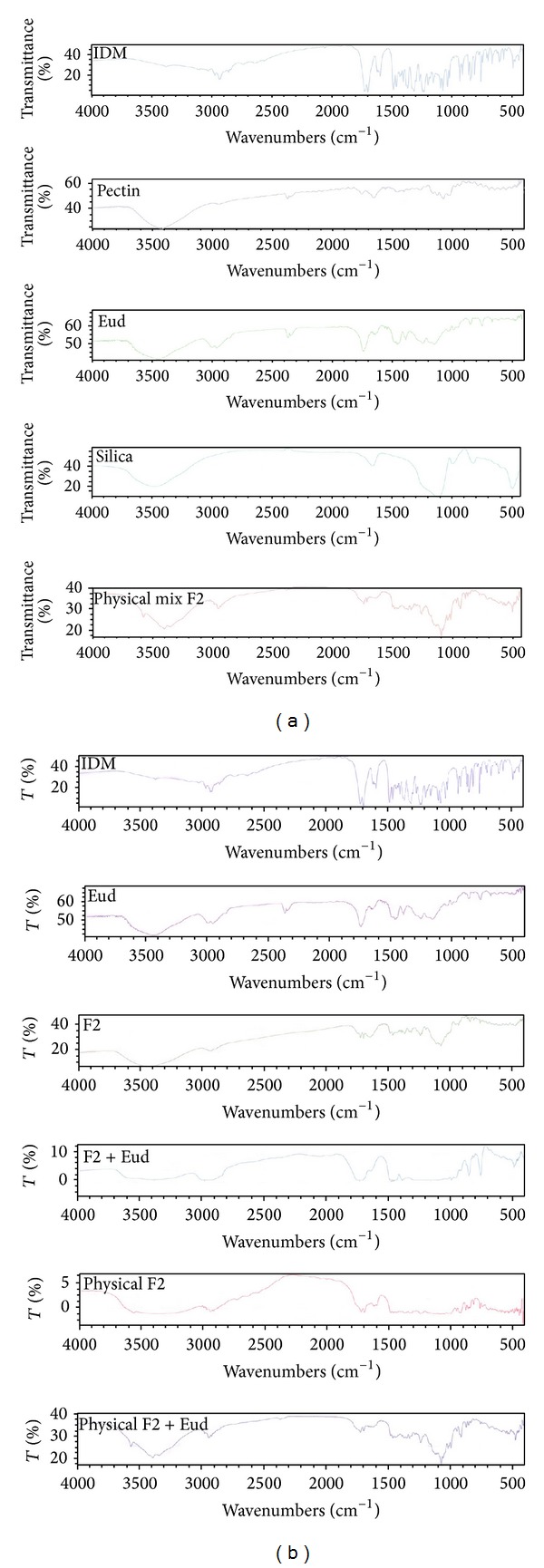
(a) IR spectra of IDM, PEC, silica, E-RL 100, and physical mixture formulation F2. (b) IR spectra of IDM, E-RL 100, formulation F2 LS, formulation F2 LS mixed with E-RL 100, formulation F2 PM, and liquisolid formulation F2 mixed with E-RL.

Dissolution profiles of IDM LSS contained in hard gelatin capsules and pure IDM containing capsule are illustrated in Figure 2. In general, all formulations showed a decrease in drug release rate in 0.1 N HCl and an increase in drug release rate in PB pH 6.8. This general observation may be attributed to the low solubility of acidic drug (pKa 4.5) in 0.1 N HCl and its high solubility in the basic medium (pH 6.8). Release characteristics are summarized in Table 3 which includes the rate of drug dissolution from all formulations after 30 min (DR 30 min) and its corresponding percentage of dissolution efficiency (DE% 30 min) in both acidic and alkaline mediums. Release profiles of IDM from all formulations F1–F6 in 0.1 N HCl and PB pH 6.8 are illustrated in Figure 3. It is obvious that CH-based LSS (F3) showed the highest drug release rate with DE% of 22.68 after 30 min of dissolution experiment in acid medium, whereas PEC-based LSS (F2) exhibited lower drug release rate in acidic medium with DE% of 15.13, after the same time of dissolution test in pH 1.2. Guar gum-based LS system (F1) exhibited the least drug release rate in pH 1.2 with DE% of 12.77 after 30 min of the dissolution test. When LSS of GG came into contact with the dissolution medium, they took up water and swelled, forming gelled particles. Then the dissolved drug diffuses out of the swollen, gelled GG [36, 37].

It has been suggested that gel formation is caused by hydrogen bonding between free carboxyl groups on the PEC molecules and also between the hydroxyl groups of neighboring molecules [38]. At low pH, ionization of the carboxylate groups is suppressed and this results in a reduction in hydration of the carboxylic acid groups and reduced number of negative charges. This decrease in the number of negative charges not only lowers the attraction between PEC and water molecules but also lowers the repulsive forces between PEC molecules themselves. Therefore, polysaccharide molecules no longer repel each other over their entire length, and, as a result, they can associate and form a gel which reduces drug release [38].

Chitosan, being basic with pKa of 6.2–7, dissolves in acidic pH showing higher drug release in 0.1 N HCl. Therefore, F3 (CH-based LSS) showed higher drug release rate than F1 and F2 after 2 hours of dissolution in pH 1.2 [39].

In PB pH 6.8, it was observed that the drug release rate depended on the extent of polymer hydration, swelling, gelling, and erosion. Guar gum gives pH-independent drug release due to its nonionic nature. It is not affected by ionic strength or pH [40]. As a result, high drug release rate in PB pH 6.8 from GG-LSS (F1) was observed. An amount of 94.00% of drug content was released with a DE% of 23.58 after 30 min of dissolution experiment in buffer pH 6.8. In case of PEC, in pH 6.8, most of the unesterified carboxyl groups are ionized producing a negative charge on the molecule which, together with the hydroxyl groups, causes it to attract layers of water. The repulsive forces between these groups, due to their negative charge, can be sufficiently strong to prevent the formation of a gel PEC network [38]. As a result, an increase in drug release rate was noticed from PEC-based LSS of IDM (F2) with a DE% of 23.66 after 30 min of dissolution test in pH 6.8. Chitosan, in pH 6.8, underwent hydration, swelling, and gelling which was followed by slow erosion and reduction in drug release from its LS system (F3). It can be seen that an amount of 67.10% of drug content was released with a DE% of 20.07 after 30 min of dissolution test in pH 6.8.

The effect of mixtures of different hydrophilic polysaccharides upon IDM release from the LSS was also studied. Drug release rate from LSS containing mixtures of GG and CH in different ratios (1 : 1, 3 : 1, and 1 : 3) was studied. Guar gum and CH mixture in the ratio of 1 : 1 showed increased drug release rate in acidic and alkaline medium compared to drug release from F1 or F3 LSS alone. On the other hand, GG : CH mixture in the ratio of 3 : 1 LSS showed the highest retardation of drug release rate (the results are not included). Formulation F4 (GG : CH mixture in the ratio of 1 : 3) was selected for further study as it showed a reasonable drug release rate retardation depending on the extent of hydration, swelling, and gelling of each hydrophilic polymer.

On using PEC and CH as a mixture for LS system (Figure 2 and Table 3), it can be seen that the percentage of drug release from LSS F5 (containing the two polymers in the ratio of 1 : 1) was higher than F2 (containing PEC alone) and lower than F3 (containing CH alone) in pH 1.2. This can be explained on the basis that CH promotes the drug release in acidic medium due to its dissolution in acidic pH. On the other hand, in PB pH 6.8, ionization of carboxyl groups increased the repulsion force due to their negative charges, thus preventing the formation of a PEC network resulting in an increase in drug release rate. Increasing the ratio of PEC in formulation (F6) by 3-fold reduced drug release rate in both pHs. The increased ratio of PEC resulted in the formation of more dense gel upon hydration which extended time to erode and dissolve.

#### 3.3.6. Consideration for Selection of E-RL 100

Different approaches have been studied for targeting drugs to the colon. The main systems are pH-based, time-dependent, and bacterially degradable. However, due to variations in physiological conditions of patients, one system alone could not be completely reliable on colonic drug delivery. Therefore, researches have been notably performed with a combination of the aforementioned systems.

Most of the commercially available systems for colon-specific drug delivery utilize Eudragit polymers (i.e., L100 and S100), soluble at pH 7, or cellulose acetate phthalate, dissolving at pH 6 as enteric materials. Eudragit RL and RS polymers have been proposed in various studies for colon targeting because of their ammonium groups content allowing a low solubility in gastric fluids [41, 42].

Eudragit RL 100 is cationic copolymer of methacrylate with quaternary ammonium groups. It is inert resins, insoluble at physiologic pHs and have pH-independent swelling properties. It is compressible and erodible and due to the presence of 10% quaternary ammonium group the Eudragit matrix is permeable [43].

Eudragit RS (E-RS) 100 and Eudragit RL (E-RL) 100 were used to produce microcarriers for a specific colon release of budesonide for the treatment of inflammatory bowel diseases [44].

The objective of another study was to produce and evaluate a novel colon-specific drug delivery system of 5-aminosalicylic acid matrix pellets using a mixture of Eudragit RS and Eudragit RL as time-dependent polymers and pectin as a bacterially degradable polysaccharide [45].

#### 3.3.7. Consideration for Selection of Liquisolid Systems

Liquisolid system containing GG (F1) showed the lower drug release rate compared to the other systems as well as the pure drug. Preliminary trials revealed that GG formed dense and thick gel that decreased the drug release from the compressed matrix. Therefore, F4 containing mixture of GG and CH in the ratio of 1 : 3 was selected to modulate drug release rate from pure GG formulation (F1). Formulation F2 containing PEC was selected since PEC is anionic polysaccharide, insoluble in acidic medium but soluble in alkaline medium. Chitosan containing LS system (F3) was selected to test the efficacy of E-RL in inhibiting the drug release in 0.1 N HCl, since CH is cationic polymer that is soluble in acidic medium. F5 and F6 containing mixture of PEC and CH in the ratios of 1 : 3 and 3 : 1, respectively, were also selected as they could modulate the drug release rate from pure PEC formulation (F2) and pure CH formulation (F3).

The obtained results indicated that all the prepared LSS had poor flow characteristics which limited its formulation into tablet dosage form unless other materials could be incorporated to improve the flow properties. In addition, due to the high solubility and swelling properties of polysaccharides in aqueous media, their dosage forms are unable to prevent the release of drugs during their transit through the stomach and the small intestine.

Therefore, a number of studies have been conducted to use a combination of polymethacrylates and polysaccharides in order to prepare more suitable dosage forms for targeting drugs to the colon [45–48]. Based on the above, each of the selected LS formulations was mixed with the appropriate amounts of E-RL 100 and lubricant and then compressed into tablets.

## 4. Precompression Evaluation

### 4.1. Fourier Transform-Infrared Spectroscopy (FT-IR) Study

The pure drug (IDM) and the solid admixture of drug and various excipients used in the preparation of matrix tablets were characterized by FT-IR spectroscopy to test their compatibility. The spectra of PEC-based formulations were included as examples. FT-IR spectra of IDM and its physical mixtures (PM) with PEC, silica, and E-RL 100 are demonstrated in Figure 3(a). All characteristic peaks of IDM are present in their original position denoting the absence of any possible interaction between IDM and E-RL or pectin. The characteristic peaks of IDM at 3370.33 cm^−1^ and 1717.14 cm^−1^ corresponding to carboxylic O−H and C=O stretch, respectively, were detected in the PM. It was also observed that the intensity of the peaks in FT-IR spectra of PM containing IDM and excipients was slightly reduced which might be due to the adsorption of the drug on more or less amorphous excipients. In general, it can be noticed that the FT-IR spectra of PM containing IDM, PEC, E-RL, and silica are superimposed with the characteristic peak of IDM.  Figure 3(b) illustrates the FT-IR spectra of PEC, E-RL, PEC-based LSS loaded with IDM (F2), and mixture of F2 and E-RL used for tableting. It was noticed that the peaks presented the sum of the peaks of IDM and PEC in F2 formulations. FT-IR spectra of the mixture of F2 and E-RL show, in addition to the F2 formulation' peaks, the characteristic peaks of E-RL. Thus, it can be concluded that there was no interaction between the content of LSS F2 and E-RL.

### 4.2. Flow Characteristics of Tablet Formulations

Although the selected LSS for preparation of tablets exhibited the lower drug dissolution rate in pH 1.2, yet they showed bad flow properties. In general, addition of E-RL 100 to the liquisolid systems of IDM improved its flow characteristics; also, addition of Mg.st : talc in the ratio of 1 : 9 as lubricant resulted in a further improvement in flow characteristics of the selected formulations (Table 6).

## 5. Postcompression Evaluation

### 5.1. Drug Content of Matrix Tablets

It was observed that all the prepared tablets complied with the test of drug content uniformity according to the USP, in which each individual content was between 85% and 115% of the average content.

### 5.2. Morphological Examination of Matrix Tablet

**Figure 4:**
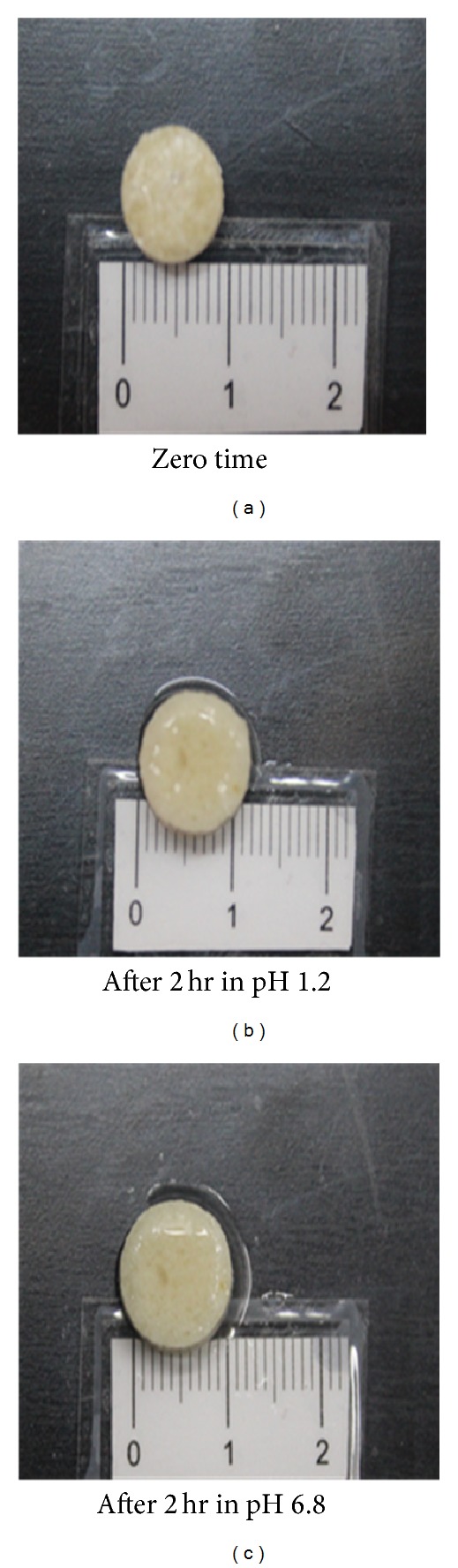
Matrix tablet of IDM-PEC liquisolid system (F2).

Visual observation indicated that the tablet matrices appeared to swell and a viscous gel mass was created when they came into contact with the medium. The degree of swelling with the formation of continuous and homogenous gel layer around the matrix was related to the hydration of each biodegradable polymer or combination of polymers in different pHs. Morphological examination offormulation F2 was only included (Figure 4). In the case of hydration of PEC matrix tablets (F2) in acidic medium (pH 1.2), the outer hydrated surface layer formed around the tablets could be seen visually to possess a very different consistency from that of the tablets hydrated in alkaline medium (pH 6.8). The hydrated layer (in acidic medium) was not viscous and adhesive in nature but represented a tough and more or less regularly swelled surface (Figure 4(a)). This is probably due to the fact that PEC is rapidly converted to pectinic acid, at pH 1-2, which has the ability to swell on hydration being virtually insoluble but it has lower swelling capacity compared to PEC [47]. In addition, it has been proved that gelation of high methoxylated PEC usually occurs at pH < 3.5 and its swelling was related to the formation of hydrogen bonds between the hydrophobic groups of polysaccharide chains [47]. Based on the above, PEC in acidic medium swells forming insoluble gel which reduced drug release rate in gastric environment.

Slight increase in tablet diameter was noticed which may be due to the swelling of both E-RL and pectinic acid. Swelling of E-RL did not form homogenous gel layer around the matrix tablet. In general, no disintegration of tablet matrices was observed indicating that the adhesive force existing between the components of the tablets was high enough to prevent tablets disintegration in both acidic and alkaline media. These results are not in agreement with the work done by Ofokansi and Kenechukwu [48]. They concluded that E-RL 100 exerted significant effect on the force of adhesion of the tablet ingredients, thereby increasing the disintegration time.

**Figure 5:**
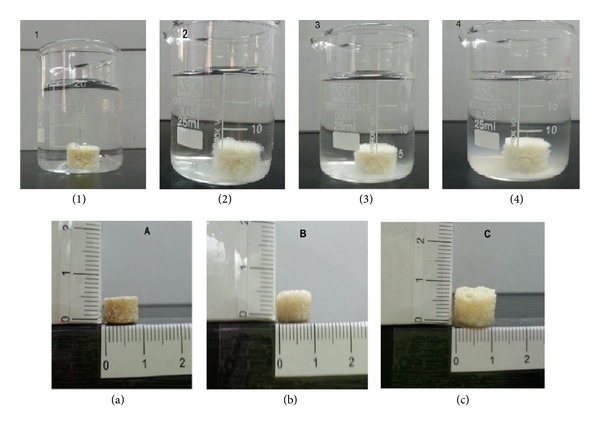
Swelling of F2 matrix tablet in pH 1.2 and 6.8 (1) at zero time, (2) after 2 hours in pH 1.2, (3) after 4 hours in pH 6.8, and (4) after 24 hours in pH 6.8. (a) Matrix tablet thickness and morphology at zero time, (b) after 2 hours in pH 1.2, and (c) after 24 hours in pH 6.8.

Figure 5 shows the swelling behavior of F2 matrix tablet in pH 1.2 for 2 hours and in pH 6.8 for 24 hours. It is obvious that, once the matrix was placed in the dissolution medium, swelling of high methoxylated PEC occurred which was manifested by the white gel network on the tablet. By time, it was observed that the gel formed did not form a continuous layer around the matrix tablet. The gel network entrapped the drug and prevented its release in 0.1 N HCl. Eudragit polymer present on the surface did not swell as the quaternary ammonium groups reacted with the chloride ions of the dissolution medium [46]. In pH 6.8, almost all the tablet matrices appeared to be covered with continuous layer of gel due to hydration and swelling of PEC and E-RL. Erosion of PEC gel network took place after 2 hours as manifested by the white cloud present in the beaker. Both intensity and magnitude of the white gelatinous cloud increased by time; the maximum erosion and dissolution were attained after 24 hours.

### 5.3. In Vitro Release Study

**Figure 6:**
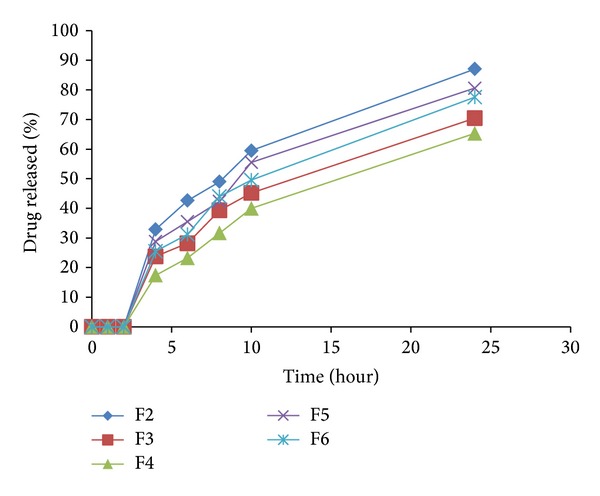
Release profiles of IDM from sustained release matrix tablets prepared from the liquisolid formulations: F2–F6.

Release profiles of IDM from all formulations are presented in Figure 6. In general, IDM release from the different tablet matrices was blocked in pH 1.2. The retardation effect of the insoluble E-RL polymer on IDM release rate from the different formulations is a common factor. The second common factor is the low pKa (4.5) of IDM and hence its solubility in pH 1.2.

The polymer side chain of E-RL 100 contains quaternary ammonium groups which is hydrophilic and facilitates the interaction of the polymer with water and thereby increases its permeability and allows the water to permeate the drug core in a controlled manner. The permeability and water uptake of acrylic polymers containing quaternary ammonium groups are influenced by the different counterions present in the medium. In acidic medium, polymethacrylates polymer contains a positively polarized quaternary ammonium group which is attracted by the negative hydrochloride counterions. Thus, the degree of polymer swelling and related drug release rate were functions of the chloride counterion interaction with the polymer's quaternary ammonium groups. In the presence of extra chloride ion, possibility of ion-exchange needed for hydration would be more restrictive [46]. In all tablet matrices, degree of swelling and permeability of E-RL in acidic medium could be extremely lowered or even prevented resulting in decrease in drug release rate.

The presence of PEC in F2 matrix tablet potentiated the retardation effect of E-RL on drug release. This finding could be explained by the fact that PEC contains carboxylic functional groups which are unionized, in pH 1.2, forming a gel network which acted as a diffusion barrier for drug release. Thus, the synergetic effect of both polymers inhibited the drug release in the acidic medium.

On immersing tablet matrix of CH into the acidic medium, free amino groups of CH got protonated and their hydration increased the degree of matrix swelling. Full ionization of all amino groups turned it into a polyelectrolyte with a relatively high charge density. In this system, the electrostatic repulsion of free ionized amino groups is responsible for swelling [48]. In addition, in acidic medium, the protonated carboxylic groups of E-RL (weak polyacid) become charged by ionized amino groups of CH to form interpolymers interaction. This polymer interaction prevented erosion of CH in pH 1.2 and inhibited drug release [48].

Matrix tablet of F4 formulation also did not show drug release in acidic pH. This is attributed to the hydration of GG by coming in contact with the dissolution media and its subsequent gel formation on the matrix tablet surface and within the matrix itself. The produced gel presented a barrier against drug molecules diffusion from the matrix in spite of the increased solubility of chitosan in acidic medium [49].

In case of tablet matrices of formulations F5 and F6 containing PEC and CH in the ratios of 3 : 1 and 1 : 3, respectively, there is no possibility of electrostatic interaction between PEC and CH as the former is present in the unionized form in acidic medium. But PEC formed a stable gel when the matrix was immersed in the acidic pH. On the other hand, as discussed before, the ionized amino groups of CH interacted with the protonated carboxylic groups of E-RL. The behavior of both polymers in acidic medium prevented drug release in pH 1.2.

**Figure 7:**
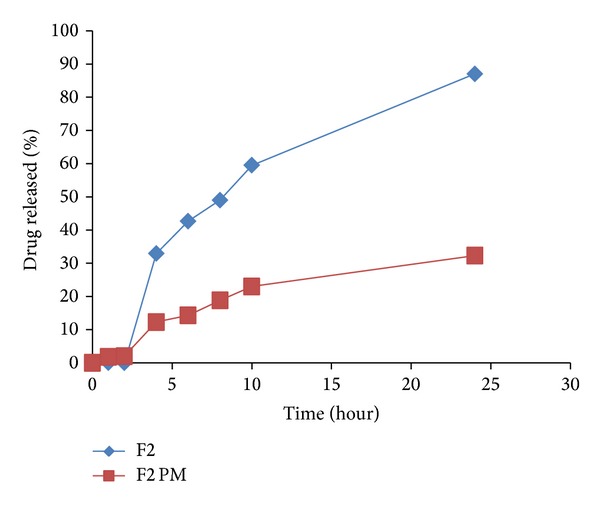
Release profiles of IDM from sustained release tablets F2 and its corresponding compressed tablet of the physical mixture F2 PM.

**Figure 8:**
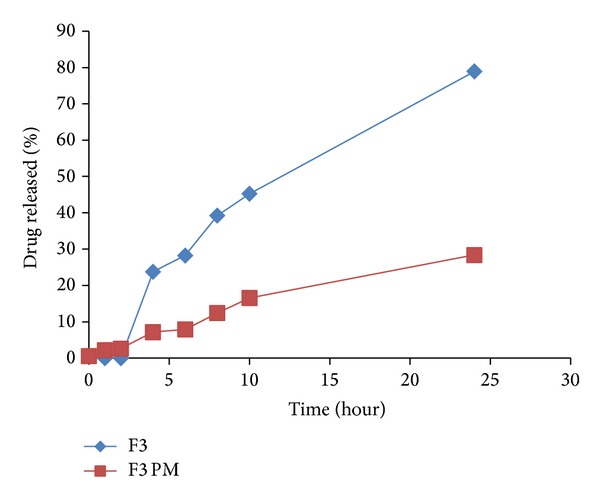
Release profiles of IDM from sustained release tablets F3 and its corresponding compressed tablet of the physical mixture F3 PM.

**Figure 9:**
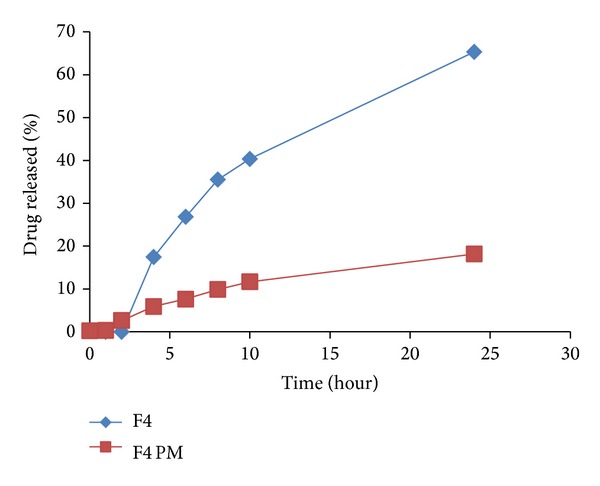
Release profiles of IDM from sustained release tablets F4 and its corresponding compressed tablet of the physical mixture F4 PM.

**Figure 10:**
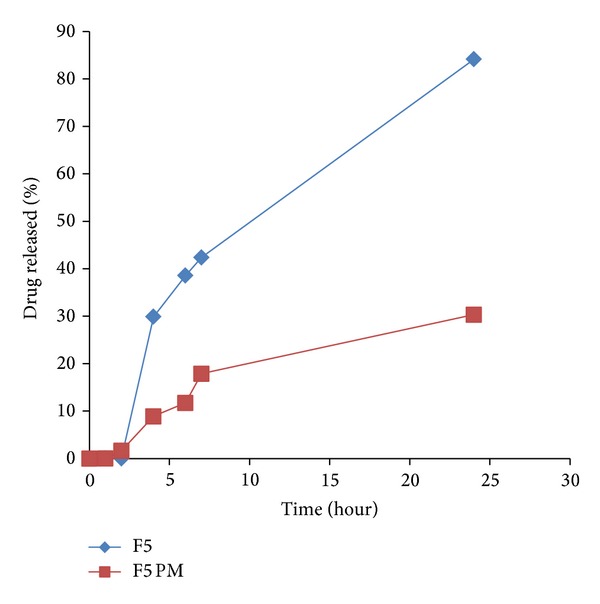
Release profiles of IDM from sustained release tablets F5 and its corresponding compressed tablet of the physical mixture F5 PM.

**Figure 11:**
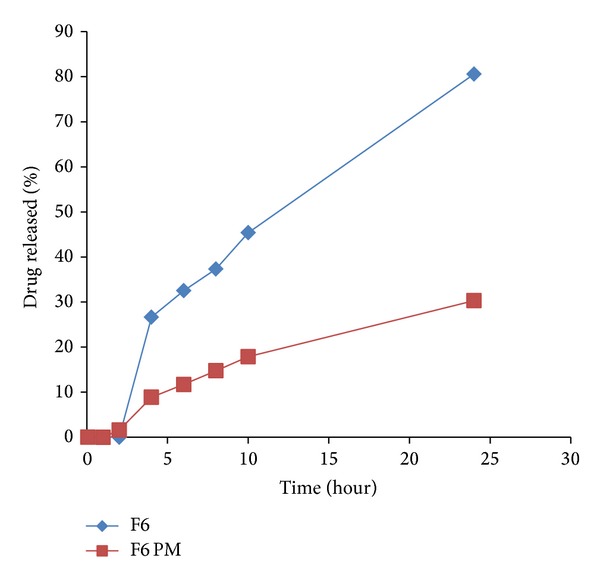
Release profiles of IDM from sustained release tablets F6 and its corresponding compressed tablet of the physical mixture F6 PM.

Drug release from all the tablet matrices in pH 6.8 is shown in Figures 7, 8, 9, 10, and 11. On transferring the matrix to dissolution medium of pH 6.8, carboxylic groups of E-RL became more ionized giving rise to an increase in the degree of swelling. On the other hand, the quaternary ammonium groups of E-RL became free from their interaction with chloride ions. The amino groups began to lose their charge and may be responsible for the increase in the hydrophobic units. As a result, the swelling slightly decreased in alkaline medium [46]. The net results denoted that permeability of the tablet matrices for dissolution medium increased, resulting in drug release in pH 6.8. Release data did not show rapid burst drug release in PB as E-RL took time for ion exchange and for the erosion of the gel formed earlier in acidic medium.

It is clear that F2 matrix exhibited the highest drug release as 32.89% of IDM was released after 2 hours of dissolution test in pH 6.8. However, F3, F4, F5, and F6 released 23.79, 17.41, 29.88, and 26.66% of their drug content, respectively, after the same time. After 2 hours of release experiment, an approximately linear sustained drug release occurred which could be due to the action of the time-dependent E-RL polymer and the hydration and swelling characteristics of the polysaccharide incorporated in the matrices. Highest IDM release from F2 matrix tablet was attributed to hydration and swelling of both E-RT and PEC in buffer medium. The permeability of the matrix increased and facilitated the penetration of dissolution medium to the inside of the matrix. In addition, according to FT-IR spectra (Figures 3(a) and 3(b)), there was no possible interaction between the polymers which could affect drug release [46].

Matrix tablet of LS formulation F3 showed a decrease in percentage of drug release rate (23.79%) compared to F2 (32.89%). This may be attributed to the decreased solubility of CH in higher pH values [48]. However, F4 matrix tablet showed the least drug release rate as about 17.41% of the drug content was released after 2 hours in pH 6.8. This may be attributed to the slow erosion of GG gel previously formed in acidic medium, the slight swelling of E-RL in pH 6.8, and the decreased solubility of CH in higher pHs. In addition, it has been reported that there is a polyionic interaction between CH and GG molecules which retarded the hydration ability of these polymers and reduced the rate of erosion of the matrix [49].

On transferring to pH 6.8, matrix tablet F5 showed higher drug release than F6. The increase in drug release rate from F5 may be explained by the inclusion of higher percentage of PEC which is soluble than chitosan in alkaline medium.

The release data revealed that all formulations succeeded to sustain drug release over a period of 24 hours. Percentage of drug release of 87.03, 70.47, 65.31, 80.59, and 77.54 was obtained from F2, F3, F4, F5, and F6 matrices, respectively. Statistical analysis of release data indicated that the difference in drug release rate was not significant (*P* > 0.05) between the five different sustained release matrix tablets.

To emphasize the efficacy of LS system to improve IDM release from controlled colon targeted matrices, tablets prepared by direct compression of the physical mixtures of all the matrix components were subjected to in vitro release studies. The release profiles are illustrated in Figures 7–11. It is obvious that the drug release from matrices containing the IDM-LS systems was much higher than that incorporating IDM powder in physical mixtures. For example, F2 matrix tablets released 42.67% of its drug content after 4 hours of release test in pH 6.8, whereas F2 conventional tablet released only 14.32% after the same time. The results of statistical analysis indicated that there was significant difference (*P* < 0.05) in drug release rate between matrix tablets prepared using liquisolid systems and those prepared using physical mixture.

## 6. Kinetics of Release Studies

It was observed that the in vitro release profiles of drug from all these formulations can be best expressed by Higuchi equation as the correlation coefficients showed the higher values (*R* ^2^: 0.996 for F4 to 0.993 for both F3 and F5) (Table 7). Higuchi's kinetics explains why the drug diffuses at a comparatively slower rate (0.053 for F2–0.060 for F3) as the distance for diffusion increases. All the formulations showed slope (*n*) values ranging from 0.557 for F3 to 1.84 for F2. The *n* values for formulations F3 indicated non-Fickian-diffusion which refers to a combination of both diffusion and erosion controlled-drug release. The other formulations showed *n* values higher than 0.89 indicating Super Case II which refers to relaxation and erosion of the polymeric chain [28]. Release kinetics studies of compressed tablets of PMs containing IDM showed the same results. The *n* values for formulations F14 indicated Fickian-diffusion, whereas *n* values for all the other formulations denoted Super Case II (Table 7).

At this stage of the study, it can be concluded that F2 matrix tablet is the formulation of choice as it prevented the release of IDM in the acidic medium and permitted its release in pH 6.8. Therefore, F2 matrix tablet was subjected to further in vitro release study using three different release media. The ability of matrix tablets of IDM (F2) to remain intact in the physiological environment of stomach and small intestine was assessed by conducting drug release studies under conditions mimicking mouth to colon transit. Drug release studies were carried out for 2 hours in 0.1 N HCl and then in PB pH 7.4 for 3 hours. After that, the experiment was continued up to 24 hours in pH 6.8 [49].

**Figure 12:**
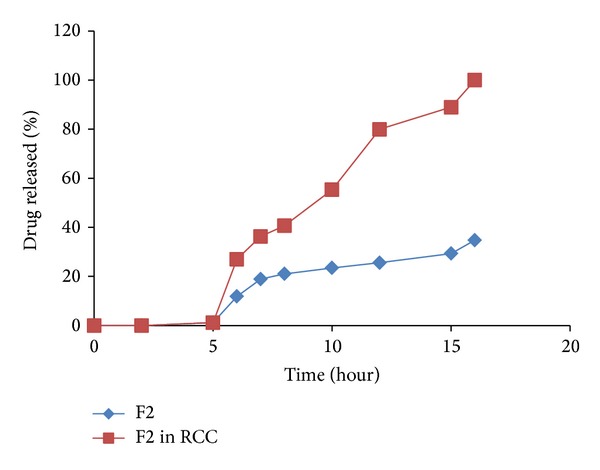
Release profiles of IDM from matrix tablet prepared from liquisolid system F2 in PB pH 6.8 and PB pH 6.8 containing rat cecal contents (2% w/v).

The release profiles of IDM from F2 matrix tablet in absence and presence of rat cecal contents are presented in Figure 12. It was found that IDM release was inhibited in pH 1.2. Inhibition of drug release in acidic medium may be attributed to the insolubility and formation of the gel network of PEC as well as the decreased permeability of E-RL 100 as discussed before. On using PB of pH 7.4, the previously gelled matrix tablets, in acidic medium, decreased the rate of penetration of dissolution medium into the tablets. The outer gelled layer increased the dissolution medium diffusion coefficient and acted as barrier for drug release. The outer layer of the tablets containing E-RL associated with the chloride ions through its protonated amino groups began to dissociate liberating protonated amino groups. The protonated ammonium groups started their charge loss, producing hydrophobic units which decreased hydration and swelling of the polymer. All these factors resulted in inhibition of drug release in pH 7.4. Near the end of the dissolution rate experiment in pH 7.4, it was found that about 0.90% of the drug content was released.

In pH 6.8 without rat cecal content, F2 matrix tablet released about 19.45% of its IDM content after one hour, whereas, in presence of rat cecal contents, percentage of drug release rate increased approximately to 27.04%. At the end of the 15-hour release test, it was observed that the drug release in presence of rat cecal contents was significantly increased compared to that in absence of rat cecal contents (*P* < 0.05). The results of release rate studies indicated that F2 LS formulation in the form of tablets succeeded to sustain drug release over a period of 24 hours, yet it failed to comply with the USP official limits of sustained drug release in absence of rat cecal content.

Drug release rate in pH 6.8 was below the official limits (of dissolution test 2) at the specified time intervals (USP). After 1, 2, 4, 12, and 24 hours of dissolution test, the sustained release matrix tablet of F2 released 11.28 (less than 15%), 18.94 (less than 35%), 22.63 (less than 55%), 25.6 (less than 75%), and 54.79 (less than 85%) of its drug content, respectively. In presence of rat cecal content in a concentration of 2% w/v, drug release rate increased to be within the official limits. It was also observed that 100% drug release rate was attained within 16 hours, which is the normal residence time of a solid dosage form in the colon. Therefore, it can be concluded that F2 matrix tablet would be considered as a promising sustained release formulation of IDM for colon delivery.

## 7. Stability Studies

**Figure 13:**
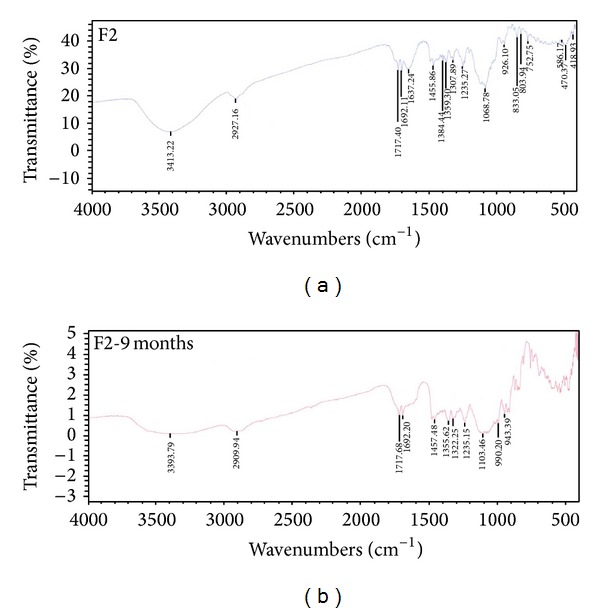
FT-IR spectra of liquisolid system (F2) before and after storage for 9 months.

**Figure 14:**
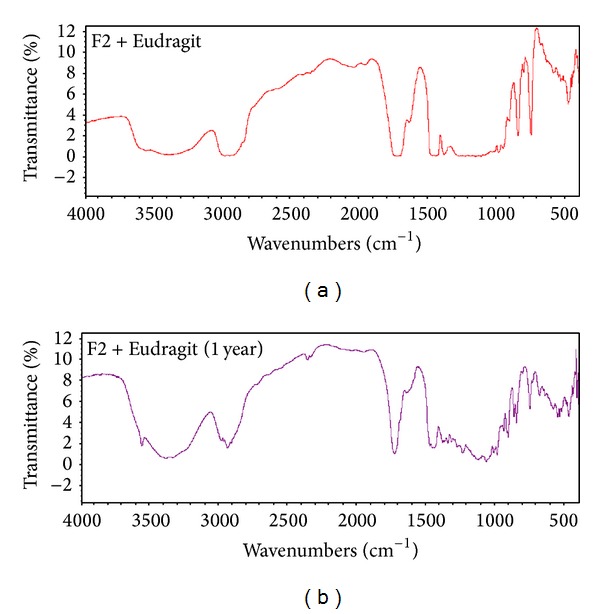
FT-IR spectra of matrix tablets (F2) before and after storage for 12 months.

Shelf stability study of LS system (F2) indicated, after 3, 6, 9, and 12 months of storage at room temperature in a desiccator, no changes in the physical appearance and drug contents of either LS system or its tablet matrix. Their release profiles before and after storage revealed insignificant change in release.  Figure 13 demonstrates the FT-IR spectra of both fresh and aged LS systems (stored for 9 months). However, Figure 14 illustrates the FT-IR spectra of both freshly prepared matrix tablet and matrix tablet after being stored for one year. It is obvious that there is no shift in the characteristic peaks of the drug indicating absence of any chemical interactions. The obtained results revealed that formulations were stable all over the period of study and could provide a minimum shelf life of 2 years [50].

## 8. Conclusion

Liquisolid technique was applied to develop successfully controlled colon-specific drug release systems. Eudragit RL 100 was employed as time-dependent polymer in combination of bacterial degradable polysaccharides which were used in the form of liquisolid systems loaded with the drug. Eudragit RL improved the flowability and compressibility of liquisolid systems. Drug release from all matrix tablets was characterized by negligible release in the initial phase followed by controlled release for a time period of 24 hours. Matrix tablet of pectin-based liquisolid system (F2) displayed a promising sustained release of the drug (in presence of rat cecal content) for 16 hours, which is the normal residence time of a solid dosage form in the colon. Liquisolid formulation F2 as well as its matrix tablets, when subjected to stability studies, indicated no significant change in physical appearance, drug content, and in vitro release pattern. Furthermore, no physical and chemical interaction was evident from FT-IR studies, indicating stability of indomethacin in the prepared matrices. An advantage of such a matrix design that comprises time-dependent polymers in polysaccharide matrices is that it can overcome the drawbacks of coated systems wherein there is a possibility of the coat remaining insoluble during its passage through the colon.

## Figures and Tables

**Table 1:** Composition of the different liquisolid systems.

Formula code	Carriers	Carriers ratio	*L*_*f*_ without IDM	*L*_*f*_ with IDM	Drug content ± SD
F1	GG	—	0.840	0.891	18.540 ± 0.028
F2	PEC	—	0.863	0.922	19.110 ± 0.028
F3	CH	—	0.887	0.947	19.670 ± 0.037
F4	GG + CH	1 : 3	0.884	0.945	19.390 ± 0.042
F5	PEC + CH	3 : 1	0.884	0.945	19.110 ± 0.030
F6	PEC + CH	1 : 3	0.863	0.922	19.670 ± 0.038

**Table 2:** Flow characteristics of IDM liquisolid formulations.

Formula's code	Polymer	Ratio of polymers	Angle of repose	Carr's index (%)	Hauser ratio
F1	GG	—	34.00 ± 0.048	19.70 ± 0.045	1.29 ± 0.002
F2	PEC	—	36.78 ± 0.052	26.66 ± 0.070	1.36 ± 0.002
F3	CH	—	40.91 ± 0.063	30.00 ± 0.057	1.42 ± 0.004
F4	GG + CH	1 : 3	39.90 ± 0.050	27.43 ± 0.057	1.39 ± 0.00
F5	PEC + CH	3 : 1	38.09 ± 0.065	27.50 ± 0.057	1.38 ± 0.003
F6	PEC + CH	1 : 3	40.70 ± 0.042	29.17 ± 0.049	1.41 ± 0.003

**Table 3:** Dissolution parameters of different formulations of IDM liquisolid compact.

Formula's code	DR_30 min⁡_ (pH 1.2)	% DE_30 min⁡_ ( pH 1.2)	DR_30 min⁡_ (pH 6.8)	% DE_30 min⁡_ (pH 6.8)
F0*	7.84	16.99	53.19	18.72
F1	3.23	12.77	94.00	23.58
F2	3.53	15.13	94.55	23.66
F3	8.70	22.68	67.10	20.07
F4	7.65	20.62	73.88	22.99
F5	4.86	21.35	80.50	22.64
F6	7.42	19.54	73.75	19.52

F0*: capsule containing 25 mg of pure drug.

**Table 4:** Composition of IDM tablets prepared using liquisolid systems and E-RL.

Formula's code	Carrier	Amount (mg)			Total weight of tablet
		LS* system	E-RL	Mg st.: talc 1 : 9	
F2	PEC	327.05	109.02	13.08	449.14
F3	CH	317.74	105.91	12.71	436.36
F4	GG + CH (3 : 1)	322.33	107.44	12.89	442.66
F5	PEC + CH (3 : 1)	327.05	109.02	13.08	449.14
F6	PEC + CH (1 : 3)	317.74	105.91	12.71	436.36

LS* system: liquisolid system equivalent to 25 mg of IDM.

**Table 5:** Composition of compressed tablets of IDM using physical mixtures.

Formula code	Carrier	Amount (mg)						Total weight of tablet
		Coat	GG	PEC	CH	E-RL	Mg st : talc 1 : 9	
F2	PEC	14.38	—	287.67	—	109.02	13.08	449.14
F3	CH	13.94			278.80	105.91	12.71	436.36
F4	GG + CH (1 : 3)	14.16	70.79		212.37	107.44	12.89	442.66
F5	PEC + CH (3 : 1)	14.38		226.53	75.510	109.02	13.08	449.14
F6	PEC + CH (1 : 3)	13.94		69.70	209.10	105.91	12.71	436.36

**Table 6:** Flow properties of mixtures of tablet components.

Formula's code	Polymer	Ratio of polymers	Angle of repose	Carr's index (%)	Hauser ratio
F2*	PEC	—	36.78 ± 0.015	26.66 ± 0.016	1.36 ± 0.016
F2 + E-RL	PEC	—	27.83 ± 0.009	21.46 ± 0.012	1.27 ± 0.013
F2 + E-RL + Mg st : talc 1 : 9	PEC	—	26.32 ± 0.017	21.09 ± 0.011	1.25 ± 0.011
F2 + E-RL (PM)**	PEC	—	25.02 ± 0.022	19.78 ± 0.012	1.13 ± 0.011
F3*	CH	—	40.91 ± 0.019	29.90 ± 0.008	1.42 ± 0.008
F3 + E-RL	CH	—	35.00 ± 0.021	21.20 ± 0.016	1.37 ± 0.016
F3 + E-RL + Mg st : talc 1 : 9	CH	—	33.10 ± 0.014	20.83 ± 0.021	1.32 ± 0.021
F3 + E-RL (PM)**	CH	—	30.20 ± 0.025	20.84 ± 0.020	1.26 ± 0.020
F4*	GG + CH	1 : 3	39.90 ± 0.009	31.67 ± 0.010	1.46 ± 0.011
F4 + E-RL	GG + CH	1 : 3	32.00 ± 0.022	21.47 ± 0.006	1.27 ± 0.007
F4 + E-RL + Mg st : talc 1 : 9	GG + CH	1 : 3	29.45 ± 0.014	21.00 ± 0.013	1.28 ± 0.012
F4 + E-RL (PM)**	GG + CH	1 : 3	26.00 ± 0.021	17.17 ± 0.021	1.20 ± 0.021
F5*	PEC + CH	3 : 1	38.09 ± 0.019	27.50 ± 0.011	1.38 ± 0.011
F5 + E-RL	PEC + CH	3 : 1	30.46 ± 0.100	21.46 ± 0.070	1.27 ± 0.070
F5 + E-RL + Mg st : talc 1 : 9	PEC + CH	3 : 1	28.99 ± 0.022	20.32 ± 0.009	1.24 ± 0.009
F14 + E-RL (PM)**	PEC + CH	3 : 1	28.50 ± 0.120	18.77 ± 0.013	1.21 ± 0.012
F6*	PEC + CH	1 : 3	40.70 ± 0.021	29.00 ± 0.011	1.41 ± 0.011
F6 + E-RL	PEC + CH	1 : 3	33.90 ± 0.017	21.56 ± 0.013	1.20 ± 0.013
F6 + E-RL + Mg st : talc 1 : 9	PEC + CH	1 : 3	30.45 ± 0.110	20.87 ± 0.011	1.25 ± 0.011
F6 + E-RL (PM)**	PEC + CH	1 : 3	29.46 ± 0.009	21.72 ± 0.010	1.18 ± 0.010

F*: liquisolid system.

PM**: physical mixture.

**Table 7:** Kinetics study of drug release.

Formula code	Zero-order		First-order		Higuchi model		Korsmeyer-Pappas' model	
	*R* ^2^	*k*	*R* ^2^	*k*	*R* ^2^	*k*	*R* ^2^	*n*
F2	0.919	0.221	0.980	0.374	0.995	0.053	0.996	1.840
F2 PM	0.929	0.626	0.064	0.016	0.971	0.126	0.975	0.809
F3	0.940	0.285	0.979	0.423	0.993	0.060	0.921	0.557
F3 PM	0.983	0.812	0.107	0.025	0.968	0.151	0.978	0.976
F4	0.962	0.321	0.982	0.416	0.996	0.059	0.990	1.320
F4 PM	0.927	0.115	0.416	0.077	0.965	0.216	0.978	0.964
F5	0.931	0.244	0.974	0.378	0.989	0.054	0.990	0.165
F5 PM	0.966	0.713	0.331	0.063	0.973	0.139	0.974	0.780
F6	0.940	0.258	0.977	0.381	0.993	0.054	0.989	1.560
F6 PM	0.966	0.713	0.157	0.038	0.979	0.180	0.959	0.937

F: matrix tablet prepared from liquisolid system.

F PM: matrix tablet prepared from physical mixture.

## References

[1] Chourasia MK, Jain SK (2003). Pharmaceutical approaches to colon targeted drug delivery systems. *Journal of Pharmacy and Pharmaceutical Sciences*.

[2] Sinha VR, Kumria R (2001). Polysaccharides in colon-specific drug delivery. *International Journal of Pharmaceutics*.

[3] Bussemer T, Otto I, Bodmeier R (2001). Pulsatile drug-delivery systems. *Critical Reviews in Therapeutic Drug Carrier Systems*.

[4] Chavan MS, Sant VP, Nagarsenker MS (2001). Azo-containing urethane analogues for colonic drug delivery: synthesis, characterization and in-vitro evaluation. *Journal of Pharmacy and Pharmacology*.

[5] Vemula VR, Lagishetty V, Lingala S (2010). Solubility enhancement techniques. *International Journal of Pharmaceutical Sciences Review and Research*.

[6] Sridhar I, Doshi A, Joshi B, Wankhede V, Doshi J (2013). Solid dispersions: an approach to enhance solubility of poorly water soluble drug. *Journal of Scientific and Innovative Research*.

[7] Blagden N, de Matas M, Gavan PT, York P (2007). Crystal engineering of active pharmaceutical ingredients to improve solubility and dissolution rates. *Advanced Drug Delivery Reviews*.

[8] Anjumn S, Farhan A, Yvonne P, Afzal RM (2011). Effects of ball-milling on PLGA polymer and its implication on lansoprazole-loaded nanoparticles. *Journal of Basic and Clinical Pharmacy*.

[9] Hart ML, Do DP, Ansari RA, Rizvi SAA (2013). Brief overview of various approaches to enhance drug solubility. *Journal of Developing Drugs*.

[10] Reza J (2013). Self-emulsifying drug delivery systems: a review. *International Journal of Pharmaceutical and Life Sciences*.

[11] Chella N, Shastri N, Tadikonda RR (2012). Use of the liquisolid compact technique for improvement of the dissolution rate of valsartan. *Acta Pharmaceutica Sinica B*.

[12] Spireas S, Bolton SM Liquisolid systems and method of preparing same.

[13] Spireas S

[14] Chandel P, Raj K, Kapoor A (2013). Liquisolid technique: an approach for enhancement of solubility. *Journal of Drug Delivery & Therapeutics*.

[15] Spireas SS, Jarowski CI, Rohera BD (1992). Powdered solution technology: principles and mechanism. *Pharmaceutical Research*.

[16] Javadzadeh Y, Siahi-Shadbad MR, Barzegar-Jalali M, Nokhodchi A (2005). Enhancement of dissolution rate of piroxicam using liquisolid compacts. *Farmaco*.

[17] Nokhodchi A, Hentzschel CM, Leopold CS (2011). Drug release from liquisolid systems: speed it up, slow it down. *Expert Opinion on Drug Delivery*.

[18] El-Houssieny BM, Wahman LF, Arafa NMS (2010). Bioavailability and biological activity of liquisolid compact formula of repaglinide and its effect on glucose tolerance in rabbits. *Bioscience Trends*.

[19] Javadzadeh Y, Jafari-Navimipour B, Nokhodchi A (2007). Liquisolid technique for dissolution rate enhancement of a high dose water-insoluble drug (carbamazepine). *International Journal of Pharmaceutics*.

[20] Khaled KA, Asiri YA, El-Sayed YM (2001). In vivo evaluation of hydrochlorothiazide liquisolid tablets in beagle dogs. *International Journal of Pharmaceutics*.

[21] Boudreau MD, Sohn KH, Rhee SH, Lee SW, Hunt JD, Hwang DH (2001). Suppression of tumor cell growth both in nude mice and in culture by n-3 polyunsaturated fatty acids: mediation through cyclooxygenase-independent pathways. *Cancer Research*.

[22] Elder DJE, Halton DE, Crew TE, Paraskeva C (2000). Apoptosis induction and cyclooxygenase-2 regulation in human colorectal adenoma and carcinoma cell lines by the cyclooxygenase-2-selective non- steroidal anti-inflammatory drug NS-398. *International Journal of Cancer*.

[23] Lönnroth C, Andersson M, Lundholm K (2001). Indomethacin and telomerase activity in tumor growth retardation. *International Journal of Oncology*.

[24] Higuchi K, Umegaki E, Watanabe T (2009). Present status and strategy of NSAIDs-induced small bowel injury. *Journal of Gastroenterology*.

[25] Higuchi T, Connors KA (1965). Phase solubility techniques. *Advances in Analytical Chemistry and Instrumentation*.

[26] Spireas S, Bolton SM Liquisolid systems and methods of preparing same.

[27] Luner PE, Kirsch LE, Majuru S (2001). Preformulation studies on the S-isomer of oxybutynin hydrochloride, an Improved Chemical Entity (ICE*™*). *Drug Development and Industrial Pharmacy*.

[28] Khan KA (1975). The concept of dissolution efficiency. *Journal of Pharmacy and Pharmacology*.

[29] Krishnaiah YSR, Bhaskar Reddy PR, Satyanarayana V, Karthikeyan RS (2002). Studies on the development of oral colon targeted drug delivery systems for metronidazole in the treatment of amoebiasis. *International Journal of Pharmaceutics*.

[30] Prasad YVR, Krishnaiah YSR, Satyanarayana S (1998). In vitro evaluation of guar gum as a carder for colon-specific drug delivery. *Journal of Controlled Release*.

[31] Korsmeyer RW, Gurny R, Doelker E (1983). Mechanisms of solute release from porous hydrophilic polymers. *International Journal of Pharmaceutics*.

[32] Nokhodchi A, Javadzadeh Y, Siahi-Shadbad MR, Barzegar-Jalali M (2005). The effect of type and concentration of vehicles on the dissolution rate of a poorly soluble drug (indomethacin) from liquisolid compacts. *Journal of Pharmacy and Pharmaceutical Sciences*.

[33] Basavoju S, Boström D, Velaga SP (2008). Indomethacin-saccharin cocrystal: design, synthesis and preliminary pharmaceutical characterization. *Pharmaceutical Research*.

[34] Oakenfull DG, Walter RH (1991). The chemistry of high-methoxyl pectins. *The Chemistry and Technology of Pectin*.

[36] Ahmed SI, Mohan SJ, Rao YM (2010). Modulating the release behavior and kinetic evaluation of diclofenac sodium from natural polymers. *International Journal of ChemTech Research*.

[37] Apparao P, Prabhakarreddy JV, Raju J, Shashidher B (2011). Formulation and evaluation of gum based matrix tablets of Lamivudine. *Der Pharmacia Sinica*.

[38] Sundar Raj AAS, Rubila S, Jayabalan R, Ranganathan TV A Review on Pectin: Chemistry due to General Properties of Pectin and its Pharmaceutical Uses. http://www.omicsonline.org/scientific-reports/srep550.php.

[39] Islama MMd, Masumb SMd, Rahmana MM, Mollab AIMd, Shaikhc AA, Roy SK (2011). Preparation of chitosan from shrimp shell and investigation of its properties. *International Journal of Basic & Applied Sciences*.

[40] Chaplin M (2006). *Water Structure and Behavior: Guar Gum*.

[41] Kadian SS, Harikumar SL Eudragit and its pharmaceutical significance. PharmainfoNet. http://www.pharmainfo.net/satishsinghkadian/publications/eudragit-and-its.

[42] Paharia A, Yadav AK, Rai G, Jain SK, Pancholi SS, Agrawal GP (2007). Eudragit-coated pectin microspheres of 5-fluorouracil for colon targeting. *AAPS PharmSciTech*.

[43] Nath B, Nath L, Kumar P (2011). Preparation and in vitro dissolution profile of zidovudine loaded microspheres made of Eudragit RS 100, RL 100 and their combinations. *Acta Poloniae Pharmaceutica—Drug Research*.

[44] Cortesi R, Ravani L, Menegatti E, Esposito F Ronconi E (2012). Eudragit microparticles for the release of budesonide: a comparative study. *Indian Journal of Pharmaceutical Sciences*.

[45] Akhgari A, Abbaspour M, Moradkhanizadeh M (2013). Combination of pectin and Eudragit RS and Eudragit RL in the matrix of pellets prepared by extrusion-spheronization for possible colonic delivery of 5-aminosalicylic acid. *Jundishapur Journal of Natural Pharmaceutical Products*.

[46] Akhgari A, Afrasiabi Garekani H, Sadeghi F (2009). Combination of inulin and time dependent polymethacrylates as a coating system to achieve colonic delivery of indomethacin. *Daru*.

[47] Efentakis M, Vlachou M (2000). Evaluation of high molecular weight poly(oxyethylene) (Polyox)polymer: studies of flow properties and release rates of furosemide and captopril from controlled-release hard gelatin capsules. *Pharmaceutical Development and Technology*.

[48] Ofokansi KC, Kenechukwu FC (2013). Formulation development and evaluation of drug release kinetics from colon-targeted ibuprofen tablets based on eudragit RL 100-chitosan inter-polyelectrolyte complexes. *ISRN Pharmaceutics*.

[49] Shankar SJ, Gaurav SB, Basavaraj BV (2010). Formulation and evaluation of controlled release matrix tablets of an antimicrobial drug. *International Journal of Pharmaceutical Research and Development*.

[50] Matthews BR (1999). Regulatory aspects of stability testing in Europe. *Drug Development and Industrial Pharmacy*.

